# The Burden of Microplastics Pollution and Contending Policies and Regulations

**DOI:** 10.3390/ijerph19116773

**Published:** 2022-06-01

**Authors:** Sunusi Usman, Ahmad Faizal Abdull Razis, Khozirah Shaari, Mohammad Noor Amal Azmai, Mohd Zamri Saad, Nurulfiza Mat Isa, Muhammad Farhan Nazarudin

**Affiliations:** 1 Natural Medicines and Products Research Laboratory, Institute of Bioscience, Universiti Putra Malaysia, Serdang 43400, Selangor, Malaysia; usunusi.bch@buk.edu.ng (S.U.); khozirah@upm.edu.my (K.S.); 2 Department of Food Science, Faculty of Food Science and Technology, Universiti Putra Malaysia, Serdang 43400, Selangor, Malaysia; 3 Department of Chemistry, Faculty of Science, Universiti Putra Malaysia, Serdang 43400, Selangor, Malaysia; 4 Department of Biology, Faculty of Science, Universiti Putra Malaysia, Serdang 43400, Selangor, Malaysia; mnamal@upm.edu.my; 5 Aquatic Animal Health and Therapeutics Laboratory (Aqua Health), Institute of Bioscience, Universiti Putra Malaysia, Serdang 43400, Selangor, Malaysia; mzamri@upm.edu.my (M.Z.S.); m_farhannaza@upm.edu.my (M.F.N.); 6 Department of Veterinary Laboratory Diagnosis, Faculty of Veterinary Medicine, Universiti Putra Malaysia, Serdang 43400, Selangor, Malaysia; 7 Department of Cell and Molecular Biology, Faculty of Biotechnology and Bimolecular Sciences, Universiti Putra Malaysia, Serdang 43400, Selangor, Malaysia; nurulfiza@upm.edu.my; 8 Laboratory of Vaccines and Biomolecules (VacBio), Institute of Bioscience, Universiti Putra Malaysia, Serdang 43400, Selangor, Malaysia

**Keywords:** plastic pollution, food safety, human exposure, the impact on health, policies and regulations

## Abstract

The relationship between humans and plastics has become intricate due to their versatile nature and low production cost. Plastics generation has surpassed that of other manufactured products, which, coupled with the prevailing poor waste management systems, makes it a serious problem for the terrestrial and aquatic environments as its final destination. Their extensive presence has continued to pose a significant threat, not only to the aquatic ecosystem but also to the approximately 3 billion people relying on it for their livelihood. Even more disturbing were the recent findings of these plastics in food and drinking water and the evidence of human exposure, the long-term health effects of which are largely unknown. This ubiquitous phenomenon has over time put plastics under critical observation, leading to the development of many local and international policies, resolutions, and directives aimed at addressing and reversing the menace. This review provided the first snapshot of the global and local governance strategies currently aimed at mitigating plastic pollution, their limitations, and future directions. The findings of the review revealed several aspects of microplastics (MPs) pollution to be overlooked in policy formulation, a laxity in policy implementation, and an apparent lack of indices to ascertain the impact of the regulations. Furthermore, there is currently no regulation on MPs contamination of food and drinking water and an apparent lack of funding for research into the health effects of plastics and their alternatives. This, therefore, necessitates the need for a well-coordinated approach at international and national levels to scale up these policies in all countries and translate them from paper to measurable, holistic, and realizable actions that will address all forms of plastic pollution.

## 1. Introduction

Plastics production has surpassed other man-made products, making its waste management a herculean task and its emerging pollutants a concern in terms of their potential negative effects on the environment and aquatic organisms. Societies have developed a culture of over-reliance on plastics due to their low production cost, stability, and versatile nature. Plastic industries began operating as far back as 1900 in the United States with the production of the first synthetic plastic. Thereafter, there has been a dramatic increase in the annual global production of plastics, from 1.5 million metric tons (MT) in 1950 to 359 million MT in 2018 [1]. Current cumulative plastic production has risen above 8 billion MT worldwide and is expected to progressively increase in the coming decades [2].

There is no doubt that increased plastic production has consequences, as vast amounts of the utilized plastics are released into the environment, polluting both terrestrial and aquatic ecosystems. After their usage, plastics are mostly discharged on the land [3], after which they subsequently become transferred to the aquatic environment. It is estimated that the coastal countries generate about 275 million MT of plastic, of which 4.8 to 12.7 million MT enter the ocean [4]. A United Nations (UN) estimate revealed the presence of about 51 trillion MPs in the seas, a value 500 times greater than the amount of stars in the entire galaxy [5].

**Figure 1:**
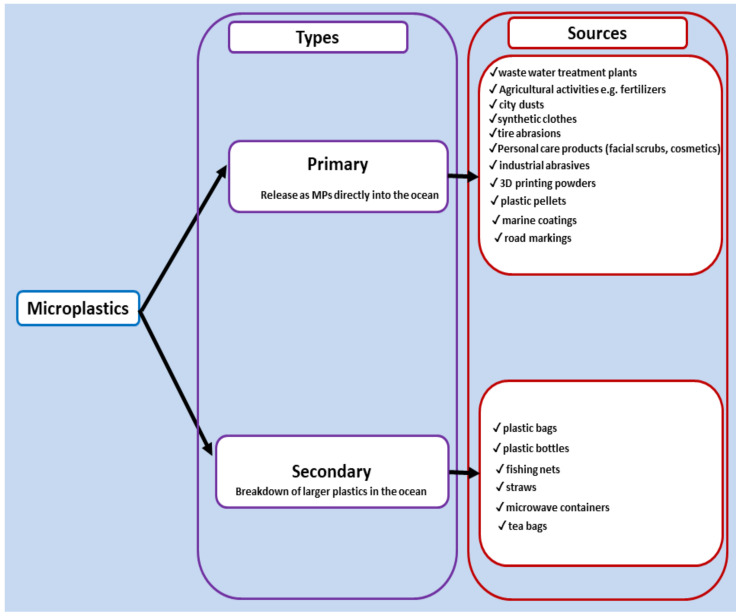
Types and sources of MPs pollution in the ocean.

MPs are a subset of plastics sized below five millimeters, whose generation and release into the environment has been projected to increase in the coming decades. As depicted in Figure 1, they are divided into two broad categories depending on their originating source. Primary MPs are released directly into the environment as small particulate materials. They account for between 15 and 35% of MPs in the oceans. The source includes MPs emanating from synthetic clothes (35%), tire abrasions (28%), and primarily synthesized MPs used as additives in personal care products such as facial scrubs (2%). About 98% of primary MPs are formed and released from land-based activities, and only 2% are generated from sea-based activities. Road runoff (66%), wastewater treatment systems (25%), and wind transfer (7%) are the major pathways that enable their accessibility into the ocean [6]. Secondary MPs contribute between 69 and 81% of MPs in the ocean and are formed following the degradation of larger plastics, such as plastic bags, bottles, and fishing nets, into smaller forms [5]. Release of MPs into the ocean from seven regions (Africa and the Middle East, China, East Asia and Oceania, Europe and Central Asia, India and South Asia, North America, and South America) showed an absolute value of between 134 and 281 kg/year with varying per capita release between regions, ranging between 110 and 170 g/person/year. The release is forecast to increase in the coming years due to an increase in the average income and inefficient MPs pollution control systems [6].

Due to their small size, MPs have become readily available to most aquatic organisms, and thus become integral part of the food web and a potential threat to food safety. Although there is documented evidence of human exposure, the long-term health implications of this are yet to be fully revealed. However, in vitro studies involving human cell lines have shown that MPs exert their effects through increased uptake, translocation, oxidative stress, and inflammation with the potential to cause metabolic diseases, neurotoxicity, and increased risk of cancer development in humans [7].

There are numerous studies using animal models that provide an insight as to the toxicity of MPs in living organisms, but their findings are yet to be exhaustive and conclusive as to whether the same effect applies to all organisms, including humans. MPs have been shown to cause serious gut flora derangement, oxidative stress, and lipid metabolism in the liver [8]. Despite the revealed negative effects on some organ systems in model organisms, the toxicity of MPs on the respiratory and endocrine systems, as well as its comprehensive toxicity mechanisms at the molecular level, is not yet understood [8]. On a general note, research on MPs-related problems is dearly needed, as less than a quarter of 192 countries have done so [9], and it is through the understanding of its deleterious effects that policies and regulations that will reverse the menace can be shaped.

With the continual presence of MPs and their effects on the environment and biota, as well as their contamination of food and the related possible health implications, plastics and MPs have been under scrutiny. This has led to the formulation of many regulations and policies by international organizations and governments of many countries around the globe. However, the policies do not focus on curbing plastic pollution from the industries down to the consumers at the final stage of its release into the environment [9].

This work highlights—albeit with some limitations—the contamination of food by MPs, and the potential health implications of this in humans. It has provided the first comprehensive review of global and local policies and regulations on plastics and their respective strategies. Furthermore, this work explores the limitations of the formulation and implementation of the policies, and provides future directions requiring further attention and evaluation.

## 2. Methodology

This review is a compilation of information obtained from scholarly, governmental, and international organizations’ databases between 2015 and 2022, using search keywords related to the topic of the review.

The methodology followed the approach of Blettler and Wantzen [10], with the objective of having a representative sample on policies, regulations, and their implementation with respect to plastics. The work has limitations and is not exhaustive, as not all information on policies and regulations may be available in the public domain.

The sourced information was duly checked and scrutinized through several stages to formulate an objective review of the topic. This involved a thorough manual checking of the results to remove papers outside the topic and repetitions, and to ensure scientific rigor [11].

## 3. Human Exposure to Microplastics

**Figure 2:**
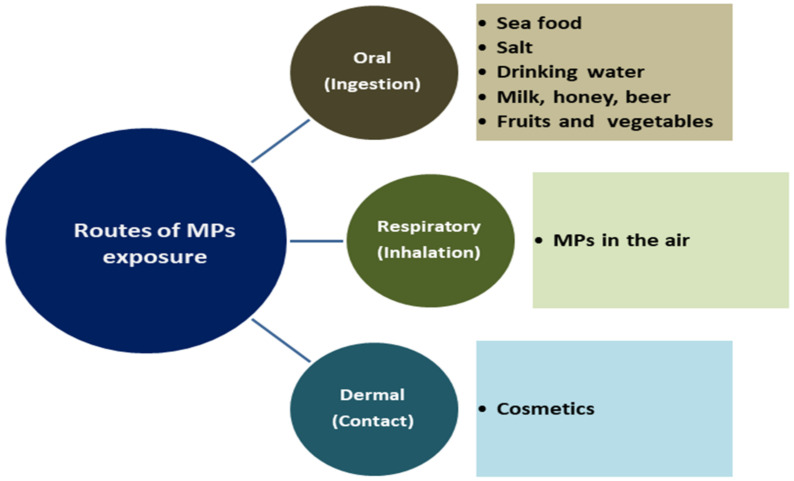
Routes of human exposure to MPs.

MPs are regarded as a major environmental health hazard; their ubiquitous nature means they have permeated all of the world’s aquatic habitats and biotas, exposing humans either directly or indirectly through various routes as shown in Figure 2, with the long-term health implications yet to be revealed. The extensive presence of MPs in coastal environments and organisms, including seafood, has raised concern about their potential dietary exposure risks in humans [10]. In addition, the epidemiology and toxicity of MPs make researching their impact on humans a priority due to the nutritional importance of seafood consumption [12].

MPs were found to be ingested and bio-accumulated in a large number of marine fish species, including those of commercial importance, and the accumulation was reported to have steadily increased, doubling over the last decade [13]. The contamination of MPs involved all the trophic levels of the food web [14]. A model for MPs accumulation has revealed a species-specific and food web-specific bioaccumulation potential, with a prediction of moderate to high bioaccumulation in lower trophic levels, indicating the health risks to marine fauna reliant on fish and coastal communities heavily dependent on seafood [15].

Although seafood remains the major exposure route through which humans ingest MPs, other sources of exposure have continued to emerge recently. The presence of MPs was observed in food and drinks, including beer, honey, and water. This may not be unconnected with the discovery of plastics particles in human stool [5,16]. However, it should be noted that despite the extensive presence of MPs in human foods, the accurate assessment of human exposure through diet is still not feasible, and will require the standardization of methods and available definitions [17]. Table 1 presents some literature examples that showcase MPs in drinking water, beverages, food, and vegetables in markets and supermarkets meant for human consumption. This has cut across many regions and countries in the world and serves as a major route through which humans become exposed to this emerging pollutant, thus posing a significant threat to human health and food safety and security.

## 4. Possible Health Impacts in Humans and Effects in Plants and Animals

It has been demonstrated that MPs exert toxicity in various capacities to virtually all biota, including plants and animals across all trophic levels in both marine and freshwater ecosystems [29]. Plants and macrophytes subjected to short- and long-term exposure to MPs were found to have various stress responses, and this has been a major concern due to their position at the bottom of the food web. They will eventually become a component of the human diet [30] and possibly exert the same or similar effect.

The long-term exposure effect of MPs on human health is largely unknown, but various mechanisms through which MPs can exert their effects in humans have been demonstrated. In support of this, an in vitro study using human cell lines has shown that MPs exert a toxic effect [7]. Due to their large surface area and persistent nature, MPs can traverse tissues and induce oxidative stress and cytotoxicity. This may enable chronic inflammation and possible cancer development. It may also increase the risk of neurodegenerative diseases and immune disorders [31]. Furthermore, chronic accumulation in the cells and tissues poses a serious hazard to humans and may result in chromosomal alterations that can ultimately lead to infertility, cancer, and obesity [32].

Plastics usually contain additives such as stabilizers and flame retardants, and, in some instances, contain toxic chemical substances that may be harmful to the animals and humans consuming them [5]. The interaction between MPs and pollutants is complex, and will largely determine how the two will have their toxic effects translated in humans and animals. The sorption and desorption processes responsible for the interaction is said to be majorly affected by the size, shape, and aging stage of the MPs, and the hydrophobic nature, functional groups, and spatial structure of the pollutants. Properties of both are heavily affected by environmental factors and alter the overall toxicity, bioaccumulation, degradation, and transportation of organic pollutants in the environment and within the organism [33]. MPs serve as vehicles for microbes, including antibiotic-resistant bacteria, which can be imported to humans through the food chain [34]. They also have the capability to absorb and accumulate persistent organic pollutants [35] and have been demonstrated to absorb heavy metals [36] and bisphenols, thereby increasing health risks [37]. Additionally, MPs were found to be vehicles for other contaminants such as pesticides, perfluoroalkyls, pharmaceuticals, polychlorinated bisphenols, and penanthrene, thereby making them emerging pollutants [38]. Furthermore, it has been postulated that MPs and plastic debris such as facemasks, sanitizers, gloves, and plastic bags are potential vehicles for SARS-CoV-2, providing an avenue for its transmission to humans and animals in the aqua-terrestrial ecosystem [39]. Although research on the interaction between metal–plastic interactions is still in its early stage, earlier studies revealed the interaction to be through weak surface interaction, with the molecular domain not yet understood. Additionally, biofilms have been shown to have the capabilities for the uptake of metal ions as micronutrients, and thus may play a key role in providing the capacity for plastics to accumulate metals [40].

The lack of knowledge as to the effects of MPs on humans has spurred an array of animal studies to explore its exposure effects on organisms. As depicted in Table 2, some examples of experimental studies using animal models have demonstrated varying degrees of effects following exposure to MPs, including inflammation, oxidative stress, tissue damage, metabolic and reproductive disorders, immunotoxicity, neurotoxicity, genotoxicity, and mortality. Whether MPs do exert the same effects on humans still remains debatable and requires further exploration to understand the underlying mechanisms and possibly extrapolate the possible health risks and biomarkers of exposure.

## 5. Efforts and Control Measures on a Global Scale by International Organizations and Regional Unions and Associations

A comprehensive review of legislations on plastics and microplastics showed a number of legislations developed to address plastics discarded in landfills. These legislations require further strengthening and review to address all forms of plastics. A review of governance strategies of controlling MPs in marine ecosystems found a lack of community involvement in monitoring and conservation, largely attributed to the absence of citizen science and co-management initiatives by key players; in addition, no standardized management strategy has been put in place [55]. The legislations heavily relied on bans, the imposition of levies, and campaigns by volunteers to ensure the reduction and reuse of plastics [56]. In addition to the need to strengthen the legislations, the review proposed a closed loop approach that integrates existing ones to shape consumer behavior, enable plastic redesign and recycling, and evaluate the impact of those reaching the landfill so as to ascertain the effectiveness of existing legislation and guide the development of new laws on single-use plastics and MPs [56].

Policies on MPs have largely neglected its pollution of agricultural land, which is mediated through sewage and plastics-coated fertilizers. This necessitates the development of policy and governance-based measures that will prevent the contamination of agricultural lands and other potential toxic elements (PTEs) that can be carried by MPs, as well as instituting regulations that will ensure food quality assurance. The measures are expected to prevent human exposure to both MPs and PTEs [57].

EU countries have recognized sewage sludge (SS) as a major factor contributing to the contamination of agricultural land, and this has led it to formulate high-level strategy for sustainable SS management by its member countries. The strategy involves multiple stakeholders being expected to work harmoniously to achieve the desired goal of appropriate and efficient management [58]. It requires a review of directive 86/278/EEC on SS that will recognize the relationship between sewage and MPs, as well strictly prohibiting SS disposal on land unless necessary. Additionally, it requires plans to actualize a circular economy and provide alternatives to SS handling through high tech processes in waste water sewage plant to be strengthened by research and development [58].

The United Nations (UN) has provided international communities with statistics detailing how plastic pollution of the oceans adversely affects marine life, and by extension the humans who largely depend on it for their livelihood. The UN has provided these statistics to guide countries on how to act. In its SDGs report of the year 2021, the UN revealed that over 3 billion people rely on the oceans for their livelihoods, and further showed the sustainability of the oceans to be under serious threat due to plastic and marine pollution, among other factors (ocean warming, eutrophication, acidification, and fishery collapse). This has led to the development of dead zones (water areas lacking sufficient oxygen to support marine life) which have increased at an alarming rate, from 400 in 2008 to 700 in 2019. It has also increased the vulnerability and lack of protection to over half of the marine key biodiversity areas.

The UN has recognized marine plastics and MPs under 13 out of its 17 sustainable development goals (SDGs) due to the pollution of the water body and the resulting adverse effects on ecosystems and livelihoods. Notable among the 13 SDGs that specifically and directly address plastic pollution is SDG number 14, which is aimed at the conservation and sustainable use of the oceans, seas, and marine resources for sustainable development. SDG 14 focuses on plastic pollution under target 14.1, which aims to prevent and significantly reduce all types of marine pollution, particularly those caused by land-based activities, by 2025. The target is expected to be measured by indicator 14.1.1b and evaluated by an index of coastal eutrophication and floating plastic debris. Only a single indicator of SDG 14 out of 247 indicators of the SDGs is meant to address the plastics problem, with the rest having no specific targets or indicators to measure their success, thus making implementation, reliable reporting, and monitoring by governments and organizations a huge challenge [59,60]. Despite this, only about half of the countries in the world have adopted initiatives to support small-scale fishermen, and on average only about 1.2% of national research budgets are allocated to ocean science [59]. Additionally, the indicator of SDG 14 to date has no internationally accepted index of floating plastic debris density.

In response to growing concerns regarding the increasing amount of marine litter—including plastics and MPs, which have become a global issue and pose serious environmental threats to marine biodiversity, ecosystems, animal health, livelihoods, fisheries, maritime transport, recreation, tourism, food safety, and the economy—the United Nations Environmental Assembly of the United Nations Environment Programme (UNEP) adopted resolutions in its fourth session on 15 March 2019, which was held between 11 March and 15 March 2019 in Nairobi, Kenya. These resolutions include the resolution on marine plastic litter and MPs (UNEP/EA.4/Res.6), which aims to control the release of plastics and MPs into the environment, provide alternatives, and halt and reverse its effects. This resolution emphasizes the need to prevent and reduce marine litter, including plastics and MPs, from land- and sea-based sources for the implementation of the 2030 agenda of sustainable development for the SDGs. It reiterates the need for sustainable management of plastics throughout their life cycle, in order to increase sustainable consumption and production patterns, including a circular economy, sustainable economic models, environmentally sound waste management, resource efficiency, the three Rs (reduce, reuse, recycle), sustainable material management, technology innovation, environmentally friendly marine plastic litter clean-up, and international cooperation to enact sustainable consumption and production patterns. It also recognizes the need to urgently strengthen science–policy interfaces at all levels so as to improve on science-based approaches that will look at the fate, distribution, and consequences of marine litter (including plastic litter and MPs) on the environment and also encourage local, national, regional, and global action to prevent and eradicate the discharge of litter, including plastics and MPs, into the marine environment [61].

The UN resolution on addressing single-use plastic product pollution (UNEP/EA.4/Res.9) was formulated due to poor management and recycling of plastic waste by all member countries in order to ensure efficient waste management and provide environmentally friendly alternatives. It was noted that less than 9% of 9 billion MT ever produced are recycled, and if plastic consumption and waste management remain as it is currently, 12 billion MT of plastics will be released in the environment by the year 2050, most of which will come from plastic packaging. These plastics are projected to heavily impact the environment through waterway blockage, clogging sewers, providing a favorable breeding ground for mosquitoes and other pests, and blocking the stomachs and airways of animals, as well as impacting on human health due to poor solid waste management practices. In an attempt to address these problems, the resolution encourages member countries to develop and implement policies to control single-use plastics at national and regional levels. It also encourages the identification and development of environmentally friendly plastic alternatives and calls for improvement in waste management that will reduce plastic waste spills into the environment. Governments are encouraged to invigorate the private sector to pursue resource-efficient design and production and also engage in educating their communities and stakeholders as to the impact of plastic pollution and the sustainable alternatives so as to promote sustainable consumption patterns. It incorporates collaboration between member states, intergovernmental and non-governmental organizations, the scientific community, the private sector, and other stakeholders to encourage research and development so as to come up with single-use plastic alternatives and also find a solution to plastic pollution at various levels. It requested funding by UNEP and other UN agencies to facilitate technical support and policies in developing countries in relation to collaboration between the government and stakeholders to enhance research into plastic alternatives and provide information as to the measures taken by the member states to address plastic pollution, all of which is to be communicated at the fifth session of the Environment Assembly [62].

The third UN resolution was formulated to control plastics pollution by integrating and implementing its resolutions with SDG and circular economy laws to ensure strict control of plastic pollution and the use of sustainable materials as alternatives. The resolution (UNEP/EA.4/Res.1) considers sustainable consumption and production as key factors for sustainable development. The resolution was passed to ensure that change in consumption and production patterns is reflected in the goal of the 2030 agenda for sustainable development through sustainable development goal 12. Its goal is to ensure the implementation of policies related to the circular economy and the use and management of sustainable materials. The resolutions 2/11 on marine plastic litter and MPs and 3/7 on marine litter and MPs are expected to address the menace of plastic pollution as part of the 10-Year Framework of the Programme on Sustainable Consumption and Production Patterns and Environment Assembly resolutions [63].

The United Nations Environment Programme (UNEP), the International Union for Conservation of Nature (IUCN), and the Life Cycle Initiative formulated guidance that provided a harmonized method expected to be used worldwide that will enable the identification of plastic leakages, referred to as “hotspots”, tracing their impacts in the plastic value chain and making provision for priority actions on the identified hotspots. The “National Guidance for Plastic Pollution Hotspotting and Shaping” provided an effective and systematic strategy and framework for countries, regions, and cities to use in their respective environments. It allows countries and regions to set a baseline benchmark to be used for assessing the progress of interventions using comprehensive, consistent, comparable, and credible-based methods that encompass existing data, tools, and resources. The guidelines are expected to significantly contribute to achieving SDG 12 (sustainable production and consumption patterns) and SDG 14 (conservation and sustainable use of the oceans, seas, and marine resources). It is also expected to aid in implementing the resolutions adopted in the fourth session of the United Nations Environment Assembly, which include but are not limited to the resolutions on achieving sustainable production and consumption, marine plastic litter and MPs, and on addressing single-use plastic production [64].

In 2019 the World Health Organization (WHO) made a call for the assessment of MPs in relation to their presence in the environment and their potential impact on human health so as to reduce pollution and prevent human exposure. It called for the reduction of plastic pollution and reiterated the need for more in-depth research to enable an accurate assessment of exposure to MPs and the implications of this on human health. It further requires the development and standardization of the methods of measuring MPs in water, studying the sources and occurrences of MPs, and testing the efficiency of different treatment processes. The WHO further required drinking water suppliers and regulators to prioritize removing chemicals and pathogens that are known to pose risk to human health, which is expected to have a double advantage, as treatment systems that are capable of removing both fecal content and pathogens will go a long way towards removing MPs effectively. It was noted that effective wastewater treatment can remove 90% of MPs, whereas conventional drinking water treatment can remove MPs of less than a micrometer. However, these will not go a long way towards providing a lasting solution to the problem, as the larger global population does not benefit from enough water and sewage treatment [65].

On 5 June 2019, the European Parliament and Council adopted a directive (EU 2019/904) in order to reduce the impact of certain plastic products on the environment and human health. It promotes a circular economy through innovative and sustainable business models, products, and materials that will lead to the efficient functioning of the internal market. The scope of the directive revolves around single-use plastic products, oxo-degradable plastic products, and fishing gear containing plastics. The directives were aimed at combatting the menace of single-use plastics in member states through consumption reduction, market placement restrictions, consumer awareness measures, and coordination measures, among others. It also directs member states to impose penalties on the infringements of national provisions adopted pursuant to the directive. An evaluation and review of the directive would be conducted by the commission by 3 July 2027, and submitted to the European Parliament, the Council, and the European Economic and Social Committee. However, the scope of the directive does not cover MPs, even though they contribute to marine litter, and the EU is expected to adopt a comprehensive approach in that respect, as currently, it only encourages producers to strictly limit MPs in their products [66].

The Association of Southeast Asian Nations (ASEAN), comprising of Brunei Darussalam, Cambodia, Indonesia, Lao People’s Democratic Republic (PDR), Malaysia, Myanmar, the Philippines, Singapore, Thailand, and Vietnam, had adopted and initiated the ASEAN Regional Action Plan for Combating Marine Debris in the ASEAN Member States (2021–2025) as a regional action which aligned with the countries’ agenda of combatting the major environmental challenge of plastics. The regions generate about 30 million tons of plastic yearly. The plan is aim to ensure a harmonious strategy that is scalable and will provide a solution to the problem of marine plastic debris in the region. The policy will align resources that will strengthen the already available actions against plastic debris in the countries and has been supported by the World Bank Group through PROBLUE, which is a trust fund under its multi donor umbrella [67]. The plan is committed to reducing plastic release into the system, increasing mop up, reducing leakage, and enhancing waste reuse by value chain creation. It has a guideline for countries that will ensure the phasing out of single-use plastics, harmonise plastic recycling and a packaging standard in the region, and enhance the capacity for monitoring and measuring marine debris in the region. The measures are expected to be coordinated and improve the capacity of the regional platform for innovation, investment, and training [67].

**Figure 3:**
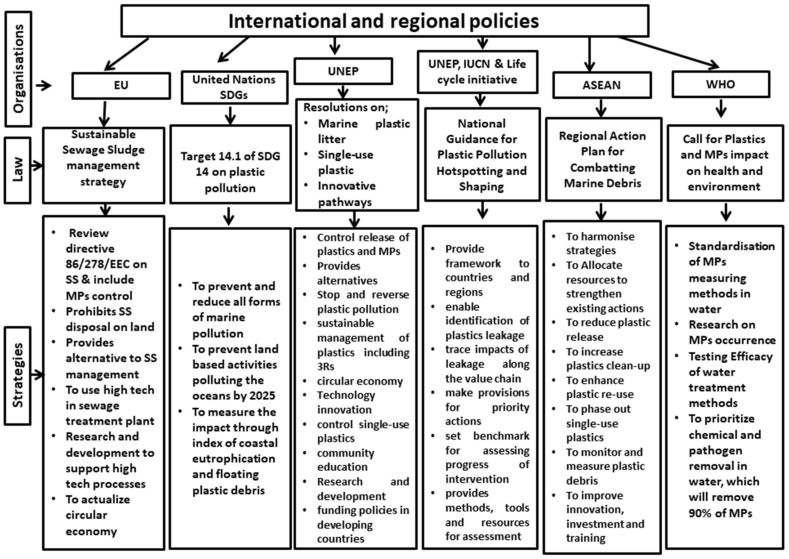
Governance strategies by international and regional organizations to combat plastic and MPs pollution.

It can be seen that, based on the foregoing discussion and as summarized in Table 3, most of the laws at the international level do not provide a framework and the tools to be utilized globally and be able to track the success of the set targets, even though they have good governance strategies as summarized in Figure 3. They depend on individual countries to interpret and devise ways to implement them, which will, in turn, depend on the country’s political will and the resources that will be allocated to address the problem. The UN has recognized the problem of plastics through 11 other SDGs in addition to SDG 14. However, allocating a single indicator out of 247 indicators to measure the impact of plastic in the ocean is highly insufficient to address the fast generation rate of plastic pollution on the planet and should be reviewed urgently. The European Parliament and Council have not included MPs in their directive, while the measures expected to address the problem by the WHO are not obtainable in most countries, despite the evidence of human consumption of MPs. This therefore requires a commitment to allocating resources that will fund research and provide realistic and measurable tools that will holistically address this problem.

## 6. Efforts and Control Measures by Countries, States, Companies, and Non-Governmental Organizations (NGOs)

Plastics have been under critical observation [68] due to their massive production and the lack of information as to their end fate, making governments, NGOs and companies put in tremendous effort through various means to address the problem. This has led to significant global improvement in policies in many countries. In the same vein, some companies, such as Toyota and Proctor & Gamble (P&G), have regulations which ensure they take full responsibility of handling and disposing of their plastic waste [69]. Furthermore, efforts by the NGOs have also been put in place, for instance the Plastic Soup Foundation and North Sea Foundation, as NGOs have provided a platform for information dissemination on microplastic containing products to allow consumers to make an informed decision [70].

In the United States, the Microbead-Free Waters Act (2005) was constituted and became effective in 2017, aimed primarily at addressing microplastics pollution and by extension that of plastic bags. The act prohibits the sale of microbeads-containing personal care products and promotes the use of biodegradable alternatives to plastics. It further enhances plastic recycling and improves the use of plastics as a synthetic crude energy source. The act also encourages the development of effective wastewater treatment plants (WWTP) to prevent the escape of MPs into the aquatic ecosystem, and also research into bioremediation technologies that will degrade MPs, such as using microorganisms [71]. The scope of the act, however, is said to be narrow and does not encourage many biodegradable options that will prevent plastic pollution in the larger environment [72]. In addition to the Microbead-Free Waters Act, state legislatures in the US have created a number of measures to reduce the prevailing usage of plastic bags in grocery stores and businesses so as to mitigate their negative impacts on the oceans, rivers, lakes, and forests, and the wildlife that inhabits them. In addition, the legislation was expected to reduce the burden on landfills and waste management.

Many states in the US have enacted legislation, most of which tries to address the problem of plastic bags by imposing a bans or levies and ensuring recycling in some instances. California, as the first state that enacted legislation in August 2014, banned single-use plastic bags state-wide at large retail stores and incurred a minimum charge of 10 cents for recycled paper. Similarly, Hawaii also had a state-wide ban on non-biodegradable plastic bags and paper bags made up of less than 40% recycled material across all its populous counties. New York banned plastic bags after the passage of Senate Bill 1508 in 2019. It is the third state to ban plastic bags in the US, with the law becoming effective in March 2020. The law will be on single-use plastic bags at grocery stores and other retailers. However, bags at meat and deli counters, bulk food areas, newspaper bags, trash bags, garment bags, and pharmacy prescription bags are exempt. An additional five states, including Connecticut, Delaware, Maine, Oregon, and Vermont, have enacted legislation banning single-use plastic bags, with Vermont placing additional restrictions on single-use straws and polystyrene containers [73].

The UK had a strategic ambition that was aimed at ensuring all plastic packages on the market were recyclable, reusable, or compostable by 2025. The strategy was in support of the commitment to leave a legacy of leaving a better environment for future generations, particularly through the ambition of zero avoidable waste by 2050 and a target of avoidable plastic waste elimination by 2042. The policies were contained in the December 2018 Resource and Waste Strategy aimed at reducing plastic waste. The policies were further followed by a series of consultations in February 2019 that came up with a number of proposals, which included a consultation on reforming the UK packaging producer responsibility system, a plastic packaging tax consultation, introducing a Deposit Return Scheme (DRS) in England, Wales and Northern Ireland, and a consultation on consistency in household and business recycling collections in England. While the packaging producer responsibility system and plastic packaging tax are UK-wide, the deposit return scheme is only in Scotland.

The UK, in addition to having its own regulations on plastics, has signed many international agreements that are aimed at reducing plastics in the marine environment, such as the Commonwealth Clean Oceans Alliance and the UN Sustainable Development Goals. It also has various obligations, such as the UN Basel Convention on the Control of Trans boundary Movements of Hazardous Waste and their Disposal (the Basel Convention) and the relevant regulations, which relate to the shipment of waste abroad, which by 1 January 2021 requires prior informed consent for the shipment of certain types of plastic waste and applies across the UK. The 2019 Manifesto commitment, which has been included in the Environment Bill 2021–2022 and will be applicable across the UK, was also put in place by the UK government to ban the exporting of plastic waste to non-OECD (Organization for Economic Co-operation and Development) countries [74].

China has plastic pollution regulations categorized under solid waste under the Law on the Prevention and Control of Environmental Pollution by Solid Wastes (LPCEPSW), aimed at to controlling plastic pollution and provision of plastics alternatives. The regulations prohibit the dumping of plastics in the aquatic environment and promote a circular economy. Additionally, there are many state laws regulating plastic waste disposal that are not effective and difficult to enforce, especially in rural communities where dumping is still on-going. This is in addition to the non-prohibition of the sale of plastic bags in markets and microbeads in personal care products [75].

The Malaysian government implemented its 2018 roadmap for zero single plastic use, meant to address and control single use plastics pollution and provide plastics alternatives through research and development. The policy was planned with set targets to span through 2030. The roadmap, if effectively implemented, will go a long way towards combating plastic pollution in the country and around the globe. This is due to Malaysia’s high plastic manufacturing capacity; the country has around 1300 plastic manufacturing factories. It is also ranked eighth out of the top ten countries with poorly managed plastic waste. The road map was aimed at setting up strong institutions to ensure the implementation of the policies in phases, whose work will include community education, use of bio bags to replace plastic bags, taxation on plastic manufacturing, pollution charges on single-use plastics, funding for R&D into environmentally friendly alternative products, and regional cooperation on marine plastic waste, among other measures. However, these measures faced many challenges, ranging from poor consumer awareness to a low plastic recycling rate. The plastic alternatives are costly and are not channeled for effective utilization after usage to trigger waste-to-wealth intervention [76]. Additionally, the policies were said to be significantly constrained in implementation due to the inconsistent application of the policy initiatives by state governments, further compounded by the lack of public knowledge and enthusiasm for household recycling [74].

Australia introduced the Recycling and Waste Reduction Bill in 2020, which incorporated an already existing Product Stewardship Act of 2011, and provided a flow chart for the country’s waste management and recycling. In addition, the country takes full responsibility of its plastic waste by banning the export of waste materials including plastic, paper, and glass [77].

Canada has published a proposed order that adds plastic products to Schedule 1 of the Canadian Environmental Protection Act of 1999 (CEPA) as a necessary regulatory step to manage plastic products. CEPA is one of the principal laws of the Canadian government that protects the environment from pollution. It is equipped with tools that address plastic pollution at different stages of its life cycle, from production, import, sale, utilization, and disposal. Additionally, the Canada-wide Action Plan on Zero Plastic Waste was released in July 2020, and is composed of coordinated timelines for improving awareness at different levels, the reduction of waste and pollution water-related activities such as fishing and aquaculture, advances in science research, plastic pollution capture and clean-up, and contribution to global action [78].

France has developed a legislative framework that regulates packaging and plastic waste under the French Environmental Code (FEC), as modified by the Law on the Circular Economy (Law No. 2020-105 of 10 February 2020—“Circular Economy Law”). It encompasses new obligations on plastic waste impacting both production and consumption habits. It has the sole aim of promoting circular economic models that are based on the eco-design of products, responsible consumption, the extension of shelf life, the reuse of products, and waste recycling. The obligations include informing consumers about products’ environmental characteristics, recyclability and repairability; the prevention of food waste and non-food products; the reinforcement and extension of extended producer responsibility (EPR); the reinforcement of obligations on waste management and sanctions on illegal waste dumping; and the objective of ending in the French market by 2040 all single-use plastic packaging. The circular law strengthened existing plastics laws, such as the ban on single-use plastic checkout bags for goods packaging at sale points, which went into effect in January 2016, and the sale of disposable plastic cups and plates, which went into effect in January 2020. It did so by introducing new measures, such as additional bans on single-use plastics including straws, plastic cutlery, expanded polystyrene containers, disposable glass lids as of 1 January 2021, and non-biodegradable tea or herbal plastic bags. It also banned the importation and production of single-use plastics intended for sale or given away free. Similarly, the 1 January 2019 ban on microbead-containing products will be extended to in-vitro medical devices (as of 1 January 2024), all rinse-off cosmetics (as of 1 January 2026), and cleaning products and products that have fallen under the European Chemical Agency’s restriction proposal (as of 1 January 2027). MPs will, however, still be allowed for the manufacture of medicinal products for human or veterinary use as an exception. The law has posed significant constraints to companies, unavoidably accompanied by sanctions, mainly administrative fines. To date, however, no tax on plastics or packaging has been imposed, and there is no real deposit return scheme for packaging or plastics [79].

In order to prevent non-biodegradable plastic microbeads from entering the environment due to their potential to harm marine life and other lives higher up the food chain, including humans, the New Zealand government passed waste minimization (microbeads) regulations in the year 2017. The regulation was categorized under Section 23 of the Waste Minimization Act of 2018, and prohibited the sale and manufacture of wash-off products that contain plastic microbeads for the purposes of exfoliation, cleaning, abrasive cleaning, or visual appearance of the product. The focus of the regulation was to address wash-off cosmetics such as toothpaste, facial and body exfoliants, and abrasive cleansing products made for household, car, and industrial use [80].

The Resource Circulation Act (RCA) was enacted by the Korean government in 2016 in order to ensure overall waste management through circular economy and resource efficiency. The concept of RCA has led to the establishment of a Plastic Waste Control Plan (PWCP) in 2018, aimed at the comprehensive management of plastic waste [81]. The PWCP has specific targets expected to be achieved within the span of years 2018–2030. The goal was to reduce plastic waste generation by 50% and recycle 70% of generated plastic waste through strategies that include the re-establishment of the production and consumption structures of plastic products so as to suppress plastic waste generation; enhancing plastic recycling limits through improving the four stages of the recycling system (production, consumption, discharge, and recycling); and reinforcing the accountability of government, local government, producers, and consumers as major stakeholders and participants. At the production stage, difficult-to-recycle products are to be phased out in favor of products that can be recycled. The responsibility of the producers is also to be strengthened. The consumption aspect is expected to be implemented by minimizing the packaging and use of disposable products at the levels of distribution and consumption, respectively. The discharge stage was to ensure the clean separation and discharge of consumers, the reinforcement of public management of blind spots, and the expansion of government support for the private sector. The last stage of recycling was to lead to the expansion of the demand for recycled products, improvement of the quality of recyclable materials, and stabilization of the recycling market. The PWCP has encountered major problems in its execution, which include the design and manufacture of difficult-to-recycle plastic products, the use of disposable products, over-packaging, dependency on private companies on discharge to waste collection processes, transportation, disposal, and difficulty in maintaining the profitability of recycling industries [82,83].

Italy has enacted a plastic packaging law that was based primarily on taxing plastic products and was expected to take effect from 1 July 2021. The law has been delayed several times, most recently due to the COVID-19 pandemic, and is now expected to take effect in 2022. The tax will exempt recycled and compostable biodegradable plastics and target single-use plastics. Single-use plastics manufactured entirely from polyethylene and polystyrene will attract a tax fee of 0.45 euros per kg. The tax will be imposed on the manufacturers of plastic in Italy, Italian business purchasers of plastic goods, sellers of plastic items whose supply comes from other EU member states, and importers of manufactured goods from countries outside of the EU [84].

The Swedish parliament has approved a tax on plastic bags, commensurate with their size, to control plastic pollution. The law took effect on 1 March 2020, with the full enforcement of taxation being effective from 1 April 2020. The target of the tax was importers and producers. However, it excludes imports of fewer than 40 bags, bags meant for personal or family usage, and bags meant for continuous usage. The law, according to the Swedish government, is to prevent the spread of MPs and is also expected to fulfill the EU goal of per capita use of less than 40 plastic bags annually by 2025 [85].

A global review of national laws and regulations by countries that limit the production, importing, usage, and dumping of single-use plastics and MPs that have a great impact on the generation of marine litter showed that as of 2018, about 60% (127 out of 192) of countries have some form of laws on plastic bags specifically targeted at production, distribution, usage, trade, taxation, levies, and disposal. The regulation varies between countries in terms of its comprehensiveness and is mostly tailored towards restrictions on free retail and distribution. Specific products (plates, cups, straws, and packaging) and specific materials, such as polystyrene, were banned by 27 countries. A tax on the manufacture and production of plastic bags and plastic bag usage charges on consumers at the national level were imposed by 27 and 30 countries, respectively. Single-use plastics extended producer responsibility has been passed into law by 62 countries and includes deposit refunds, recycling targets, and product take-back. As of 2018, regulations on microbeads were imposed by 4% (8 out of 192) of countries, including Canada, France, Italy, the Republic of Korea, New Zealand, Sweden, the United Kingdom of Great Britain and Northern Ireland, and the United States of America. A proposal was made in the same vein to ban microbeads at the national level by Belgium, Brazil, India, and Ireland. However, in 7 out of the 8 countries, the laws and regulations that control the use and manufacture of personal care products include only a fraction of the personal care products that were documented to contain microbeads. It should also be noted that the report does not assess how effective the implementation and enforcement of the measures taken by these countries are [86].

In Africa, 34 out of the 54 countries have passed laws that ban plastics which are either implemented or are intended to be implemented; however, 16 of the countries have imposed laws that totally ban plastic bags without guidelines that will enable implementation of the ban [87]. Plastic bags have been a major problem in most African countries, leading many governments to include the imposition of plastic bag levies or bans as means of plastic bag waste management. However, the legislation in African countries has many challenges. For example, in Mali, Kenya, Zimbabwe, Guinea-Bissau, Ethiopia, Eritrea, Tanzania, Chad, Cameroon, Mauritania, Morocco, Niger, Somalia, Rwanda, Tunisia, Mozambique, Botswana, and South Africa there is resistance by major actors benefiting from the industry, as well as poor implementation of the laws. In Zimbabwe there is are old waste management laws that cannot cope with the current reality. Additionally, there is a lack of effective substitutes that will replace the banned plastic bag [88].

**Figure 4:**
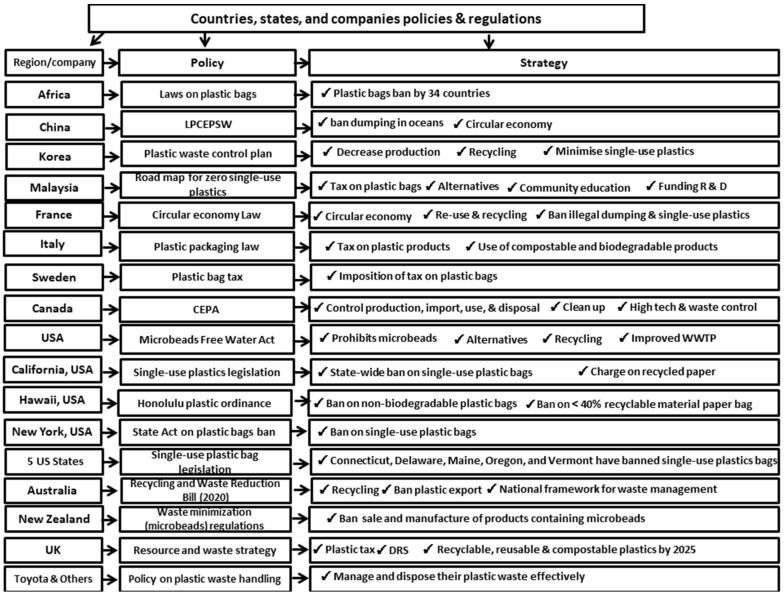
Countries, states, and companies strategies on plastics pollution control.

It can be seen that, generally speaking, there is a rising number of policies and regulations around the globe with respect to plastics and MPs, as depicted in Table 4, with summary of the strategies employed presented in Figure 4. However, there are still a considerable number of countries that either have not enacted the law or are in the process of enacting it on plastics. The implementation of the law in most instances is challenging or not enforceable in rural areas. To a large extent, the regulations still allow for the usage of plastic bags and MPs in some instances. Single-use plastics are taxed in most countries to minimize their use. However, this might not really change consumer behavior. There is a need for countries to commit certain percentages of their national budgets to encourage research and development that will allow for understanding the extent of the impact of plastic pollution, its negative impact on the ecosystem and human health, and developing plastic alternatives that will ensure a transition into the circular economy. The outputs of the research will ensure a more scientific and convincing dissemination of information that will influence the behavior of consumers and allow them to make an informed choice about using plastic products. It will also lead to the development of a cost-effective plastic alternative that can be widely used to replace plastic.

## 7. Conclusions and Recommendations

**Figure 5:**
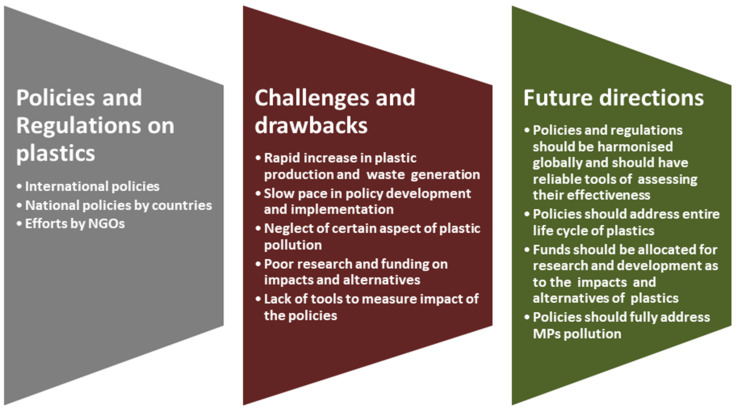
Summary of plastics policy and regulations, challenges, and future directions.

Plastic production and indiscriminate disposal have continued to increase drastically and pose serious threats to the environment, aquatic organisms, food safety, human health, and livelihood. Many policies were developed to address the problem, all of which had flaws of varying severity, which should be tackled in the future, as summarized in Figure 5. Notable among them is the slow pace of their development and implementation to cope with aggressive and rapidly rising levels of production and indiscriminate disposal. Furthermore, there is an apparent lack of effective tools to measure the impacts of the policies, in addition to not addressing the problem at every stage of the plastic’s life cycle. In most instances, not much emphasis is given to research and development in relation to their impact on aquatic organisms, food safety, human health, and plastic alternatives. In addition, the policies have overlooked certain types of plastics, such as MPs. It is therefore recommended that policies that are beautiful on paper should be aggressively expanded and harmonized at national and international levels to incorporate measurable tools that will measure their impact and also address the menace of all forms of plastic pollution holistically on a global scale.

## Figures and Tables

**Table 1:** Some examples of microplastics containing foods, beverages and drinking water in the market.

Country	Product(s)	Plastic Polymer	References
Northern Tunisia	Commercial molluscs	PE, PP	[18]
China	Bivalves	Fibres, fragments, and pellets,	[19]
China	Commercial salt	PET, PES, PE, CP, PP	[20]
Malaysia	Commercial fish	PP, PE, PET	[21]
Malaysia	Dried commercial fish	PP, PE, PET, PS, PA	[22]
UK	Commercial mussels	PP	[23]
Germany	Returnable water Single plastic bottle water and beverages	PET, PP	[24]
Mondego estuary	Commercial fish	PP, PAN, PE, polyamide 6—nylon	[25]
Catania	Vegetables and fruits		[26]
Ecuador	Milk, drinks, honey and beer	PP, HDPE, PAAm	[27]
Australia, France, Iran, Japan, Malaysia, New Zealand, Portugal and South Africa	Commercial salt	PET, PE, PP	[28]

Polyethylene; PE: polypropylene; PP: polystyrene; PS: polyethylene terephthalate; PET: Polyester; PES: Polyamide; PA: nylon; PAN: Poly 1-butene; CP: cellophane; PAAm: polyacrylamide; HDPE: High density polyethylene.

**Table 2:** Some examples of exposure effect of microplastics as demonstrated by animal studies.

Organism	Tissue	Response(s)	References
Zebra mussels (*Dreissena polymorpha*)	Gills	Modulation of proteins involved in the structure and function of ribosomes, energy metabolism, cellular trafficking, RNA-binding and cytoskeleton, all of which were related to the response against the oxidative stress	[41]
Mice	Liver	Decreased ATP, LDH and AChE Increased GSH-Px and SOD	[42]
Mice	Gut, liver	Gut microbiota dysbiosis and hepatic lipid metabolism disorders	[43]
Mice	Gut, liver	Gut damage, metabolic disorders and microbiota dysbiosis	[44]
*Mytilus galloprovincialis*	Gills, digestive glands and haemolymph	Immunotoxicity, neurotoxicity, genotoxicity, changes in gene expression profile	[45]
Hydra attenuate		Reduced feeding	[46]
Medaka (*Oryzias melastigma*)		Significant mortality, impairment of growth and egg production	[47]
*Eriocheir sinensis*	Liver	Growth inhibition, induction of oxidative stress and damage of the liver and pancreas	[48]
Earthworms (*E. foetida)*		Significant inhibition of growth and mortality	[49]
Fresh water crustacean (*Daphnia magna*)	Gut	Increased mortality and accumulation of PET in the gut	[50]
Zebra fish		Induction of microbiota dysbiosis and inflammation in the gut	[51]
Zebra fish		Induction of inflammation, lipid accumulation and oxidative stress in the liver. Additionally, there was alteration in metabolic profiles and energy metabolism	[52]
Zebra fish (*Danio rio*)		Gut inflammation, oxidative stress and significant alterations in the gut microbiome and tissue metabolic profile	[53]
Javanese medaka (*Oryzias javanicus*)	Gut, liver, kidney and brain	Histological alterations in all organs, oxidative stress and increased permeability in the gut, oxidant damage and neurotoxicity in the brain	[54]

**Table 3:** Policies and regulations on plastics by international organizations with associated challenges.

Organization	Policy/Goal	Effective	Function	Challenges/Drawback
UN	Sustainable Development Goals	2016	To conserve and sustainably use the oceans, seas and marine resources for sustainable development. It aims to prevent and reduce marine pollution, including plastics, from land activities under target 14.1, to be measured by index of coastal eutrophication and floating plastic debris [59].	There are 11 SDGs in addition to SDG 14 related to plastics pollution; however, only a single indicator of SDG 14 among 247 indicators was meant to measure the impacts of plastic pollution, which itself has no internationally acceptable index. The rest of the SDGs have no specific targets or indicators.
UNEP	Resolution on marine plastic litter and MPs	2019	To ensure long-term elimination of MPs and litter on the ocean and to prevent the ecosystems from human activities. It aims to prevent and reduce plastics and MPs from land-based activities for the implementation of SDG 2030 agenda. It entails the use of circular economy, technology innovation, and science-based approach to address the problem [61].	The three resolutions by UNEP do not have specific framework as a guide to member countries that will enable smooth and measurable implementation. It is passed and expected to be used by all member countries; however, countries will differ in using and implementing the resolutions which will depend on several factors such as existing laws on plastic waste management, presence and efficiency of waste management system, capacity to develop and utilize plastic alternatives, and overall budgetary allocation related to plastic pollution and prevention control. This will make it difficult to monitor the impact of implementation level of these resolutions, especially with the rapid and continuous release of plastics in the environment at a much higher pace than the formulation and implementation of policies which are still absent or poorly implemented in many countries.
UNEP	Resolution on addressing single-use plastic products pollution	2019	Encourages countries to develop and implement policies to control single-use plastics at national and regional levels. It also encourages the use of plastic alternatives and improvement of waste management. It requested UNEP and other UN agencies to provide funding for provision of funding and technical support and policies in developing countries [62].	
UNEP	Resolution on sustainable consumption and production	2019	It is passed to ensure change in consumption and production pattern is reflected in 2030 agenda of the sustainable development through SDG 12 [63].	
UNEP, IUCN and Life cycle Initiative	National Guidance for Plastic Pollution Hotspotting and Shaping.		A harmonized guidance expected to be used worldwide for the identification of plastic leakages by providing framework and tools to assess the progress of the intervention. It is expected to contribute in achieving SDG 12 and 14 and implementation of UNEP resolutions on marine plastic litter, single-use plastics, and sustainable production and consumption [64].	
WHO	A call for assessment of MPs presence in the environment and their impact on human health	2019	It encourages scrutiny of MPs in the environment and their human health impact. It mandates the development and standardization of methods of MPs measurement in water and directs suppliers and regulators to give priority to removing chemical pathogens from drinking water, which is expected to remove 90% of MPs [65].	The call doesn’t have any guideline and tools that will track its implementation by countries. Most of the world population does not benefit from water and sewage treatments that will address the problem of MPs in the long term.
ASEAN	ASEAN Regional Action Plan for Combating Marine Debris in the ASEAN Member States (2021–2025)		The plan is committed to reducing plastic release, increasing mop up and reducing leakage, and enhancing waste reuse by value chain creation.	

**Table 4:** Policy and strategies by countries, states, companies and non-governmental organizations.

Country/State/Organization	Policy/Law	Function	Drawback/Challenge
Africa (34 out of 54 countries)	Laws banning plastic bags	The laws impose plastic bag ban/impose levies [87,88]	Resistance by major stakeholders, poor enforcement and lack of alternatives [88].
China	Law on the Prevention and Control of Environmental Pollution by Solid Wastes (LPCEPSW)	It regulates waste dumping sites and prohibits plastic dumping in rivers, lakes, and reservoirs. It also promotes circular energy [75].	The laws are difficult to impose in rural areas. Plastic bags and microbeads in personal care products are not yet prohibited [75].
Korea	Plastic Waste Control Plan	It was aimed at reducing plastic waste generation by 50% and recycle 70% of generated plastic waste. It plans for re-establishment of production and consumption structures and circular economy [81].	It faces challenges especially in design and manufacture of difficult to recycle plastic products and continuous use of disposable products and over packaging. The waste management is highly dependent on private companies and maintaining profitability is difficult for the recycling companies [81].
Malaysia	Road map for zero single-use plastics	It instituted tax on single-use plastic bags and plastic manufacturers and set up a communication, education, and public awareness unit. It also encourages research and development on plastic alternatives such as bio bags [76].	There is poor consumer awareness, low plastic recycling rate, poor policy implementation, and a poor integrated waste management approach [74]. In addition, plastic alternatives have a high cost and there is inconsistent application of policy initiatives by states.
France	Circular Economy Law	It banned single-use plastics and promotes circular economic models [79].	MPs are allowed in medicinal products for human and veterinary use [79].
Italy	Plastic packaging law	The law imposes tax on single-use plastics, plastic manufacturers, business purchasers, and sellers of plastics in Italy [84].	The law has been delayed several times and expected to take effect in 2022. It also exempts recyclable and compostable plastics.
Sweden	Plastic bag tax	Tax was placed on importers and producers of plastic bags so as to prevent the spread of MPs and to fulfill the EU goal of per capita use of less than 40 plastic bags annually by 2025 [85].	The law has exempted import of less than 40 bags meant for personal or family use and bags meant for continuous usage [85].
Canada	Canadian Environmental Protection Act of 1999 (CEPA)	It was aimed at addressing plastic pollution using tools at different stages of their life cycle from production, import, sale, utilization, and disposal [78].	
USA	Microbeads Free Water Acts (2005)	It prohibited the sale of personal care products containing microbeads and had set up a committee to create response strategy [71].	The scope was narrow and does not encourage biodegradable alternatives that will prevent plastic pollution in the larger environment [72].
CA, USA	Legislation of single-use plastic bags	It banned all single-use plastic bags state-wide and had imposed a charge of 10 cent minimum for recycled paper [73].	
Hawaii, USA	Honolulu plastic ordinance	It was aimed at reducing single-use fossil plastics and replacing them with paper and plant-based plastic [73].	
NY, USA	State Act on plastic bags ban	It imposed a ban on single-use plastic bags at grocery stores and other retailers [73].	Plastic bags at meat/deli counter, bulk food area, newspaper bags, trash bags, garments bags, and pharmacy prescription bags were exempted [73].
Other US States (Connecticut, Delaware, Maine, Oregon and Vermont)	Legislation on single-use plastic bags	The five states have imposed ban on single-use plastic bags [73].	
Australia	Recycling and Waste Reduction Bill (2020)	It banned plastic export and provided flow chart of waste management and recycling [77].	
New Zealand	Waste minimization (microbeads) regulations	It prohibited the sale and manufacture of wash off products containing microbeads [80].	
UK	Resource and waste strategy	Aimed to ensure all plastic packages on the market were recyclable, reusable or compostable by 2025. It also imposed plastics packaging tax [74].	
Plastic Soup Foundation and North Sea Foundation	Development of MPs information Apps	It gives information to consumers and allow them to make informed choices about using products containing MPs [70].	
Toyota, Walmart, Procter & Gamble	Taking responsibility of their plastics	Disposing plastic waste to land fill and plastics recycling [69].	
